# Production of Different Biochemicals by *Paenibacillus polymyxa* DSM 742 From Pretreated Brewers’ Spent Grains

**DOI:** 10.3389/fmicb.2022.812457

**Published:** 2022-03-04

**Authors:** Blanka Didak Ljubas, Mario Novak, Antonija Trontel, Ana Rajković, Zora Kelemen, Nenad Marđetko, Marina Grubišić, Mladen Pavlečić, Vlatka Petravić Tominac, Božidar Šantek

**Affiliations:** Department of Biochemical Engineering, Faculty of Food Technology and Biotechnology, University of Zagreb, Zagreb, Croatia

**Keywords:** *Paenibacillus polymyxa* DSM 742, biochemicals, 2,3-butanediol, lactate, brewers’ spent grains

## Abstract

Brewers’ spent grains (BSG) are a by-product of the brewing industry that is mainly used as feedstock; otherwise, it has to be disposed according to regulations. Due to the high content of glucose and xylose, after pretreatment and hydrolysis, it can be used as a main carbohydrate source for cultivation of microorganisms for production of biofuels or biochemicals like 2,3-butanediol or lactate. 2,3-Butanediol has applications in the pharmaceutical or chemical industry as a precursor for varnishes and paints or in the food industry as an aroma compound. So far, *Klebsiella pneumoniae*, *Serratia marcescens*, *Clostridium sp.*, and *Enterobacter aerogenes* are being used and investigated in different bioprocesses aimed at the production of 2,3-butanediol. The main drawback is bacterial pathogenicity which complicates all production steps in laboratory, pilot, and industrial scales. In our study, a gram-positive GRAS bacterium *Paenibacillus polymyxa* DSM 742 was used for the production of 2,3-butanediol. Since this strain is very poorly described in literature, bacterium cultivation was performed in media with different glucose and/or xylose concentration ranges. The highest 2,3-butanediol concentration of 18.61 g l^–1^ was achieved in medium with 70 g l^–1^ of glucose during 40 h of fermentation. In contrast, during bacterium cultivation in xylose containing medium there was no significant 2,3-butanediol production. In the next stage, BSG hydrolysates were used for bacterial cultivation. *P. polymyxa* DSM 742 cultivated in the liquid phase of pretreated BSG produced very low 2,3-butanediol and ethanol concentrations. Therefore, this BSG hydrolysate has to be detoxified in order to remove bacterial growth inhibitors. After detoxification, bacterium cultivation resulted in 30 g l^–1^ of lactate, while production of 2,3-butanediol was negligible. The solid phase of pretreated BSG was also used for bacterium cultivation after its hydrolysis by commercial enzymes. In these cultivations, *P. polymyxa* DSM 742 produced 9.8 g l^–1^ of 2,3-butanediol and 3.93 g l^–1^ of ethanol. On the basis of the obtained results, it can be concluded that different experimental setups give the possibility of directing the metabolism of *P. polymyxa* DSM 742 toward the production of either 2,3-butanediol and ethanol or lactate.

## Introduction

A focus of the modern industry is to decrease large amounts of by-products and transform them into more environmentally friendly products (Tang et al., 2009). A good example of such industry is beer production in which by-products are available through the whole year. Two main by-products of beer production are spent yeasts (≈125,000 t/y in Europe) and brewers’ spent grains (BSG) (≈38.6 million tons worldwide) that can be used as livestock feed, or in different biotechnological processes (Karlović et al., 2020). After mashing and lautering, BSG is separated from wort, and it accounts for 85% of total waste in the beer industry which makes it a very attractive raw material for the biotechnological industry in the production of active coal and biomethane as well as extraction of high-value products. BSG is a lignocellulosic biomass that contains 40–50% of polysaccharides (starch, glucans, arabinoxylans), 30% proteins, 5% ash, and 15% of lignin. The content depends on the brewing process: sweetening and sanding, as well as cereal used in the process (Tang et al., 2009; Ikram et al., 2017; Jackowski et al., 2020). Pretreatment of BSG releases fermentable sugars which can be used as a substrate for the growth of microorganisms and the production of different biochemicals. Except being a carbon source, it is also a good source of amino acids such as tryptophan, phenylalanine, histidine, methionine, and lysine (Ikram et al., 2017). In addition, phenolic compounds from the BSG show immunomodulatory activity in inflammatory processes (McCarthy et al., 2013).

*Paenibacillus polymyxa* (formally *Bacillus polymyxa*) is a sporogenic, rod-shaped, gram-positive bacterium (Jeong et al., 2019) which is found in different habitats like soil, rhizosphere, plant tissue, and fermented foods (Meena et al., 2017). This bacterium produces a wide range of secondary metabolites like phytohormones, polysaccharides, and antimicrobial substances which are involved in adaptation and survival in different environmental conditions (Grady et al., 2016). Some of the secondary metabolites are antimicrobial lipopeptides, such as antibiotic polypeptins, polymyxins, and fusaricidins, which inhibit plant pathogens and are therefore useful in agronomy (Li and Chen, 2019). Some of the strains synthetize antibiotic polymyxin E1 which is efficient against Gram-negative bacteria, while other strains produce lantibiotic and paenibacillin, antibiotics useful against Gram-positive bacteria (He et al., 2007; Lal and Tabacchioni, 2009). Apart from the antibiotics, *P. polymyxa* produces different peptides which have strong antifungal activity against fungi such as *Aspergillus niger*, *Aspergillus oryzae*, *Fusarium oxysporum*, and yeasts *Saccharomyces cerevisiae* and *Candida albicans* (Kajimura and Kaneda, 1997). Bacterium *P. polymyxa* also promotes plant growth by atmospheric nitrogen fixation and synthesis of plant hormones (Meena et al., 2017).

*Paenibacillus polymyxa* has potential for production of biochemicals, polymers, and enzymes such as 2,3-butanediol (2,3-BD), exopolysaccharides, cellulases, glucanases, proteases, and xylanases (Lal and Tabacchioni, 2009). These products play a significant role in the agriculture, food, and biotechnological industries (Daud et al., 2019). The market value of 2,3-BD in 2020 was estimated to be around 76 million USD. It is used as a building block in different industries, such as the chemical industry for production of plastics and anti-freeze, in the cosmetic and food industries. Various bacteria, such as *Enterobacter*, *Klebsiella*, and *Serratia* sp., produce 2,3-BD with a higher yield than bacterium *P. polymyxa*. The downside of these bacteria is their pathogenicity which makes the bioprocess more complex and expensive regarding microbial safety. Additionally, they produce *S,S*-2,3-BD or *meso*-BD which is less suitable for industrial application. *P. polymyxa* is a non-pathogenic bacterium with a GRAS status which produces 98% pure optical active *R*,*R*-2,3-BD (Moo-Young, 2011). Besides 2,3-BD, *P. polymyxa* produces other biochemicals such as ethanol, acetate, lactate, formate, and succinate which can also be very valuable biotechnological products (Hakizimana et al., 2020).

**FIGURE 1:**
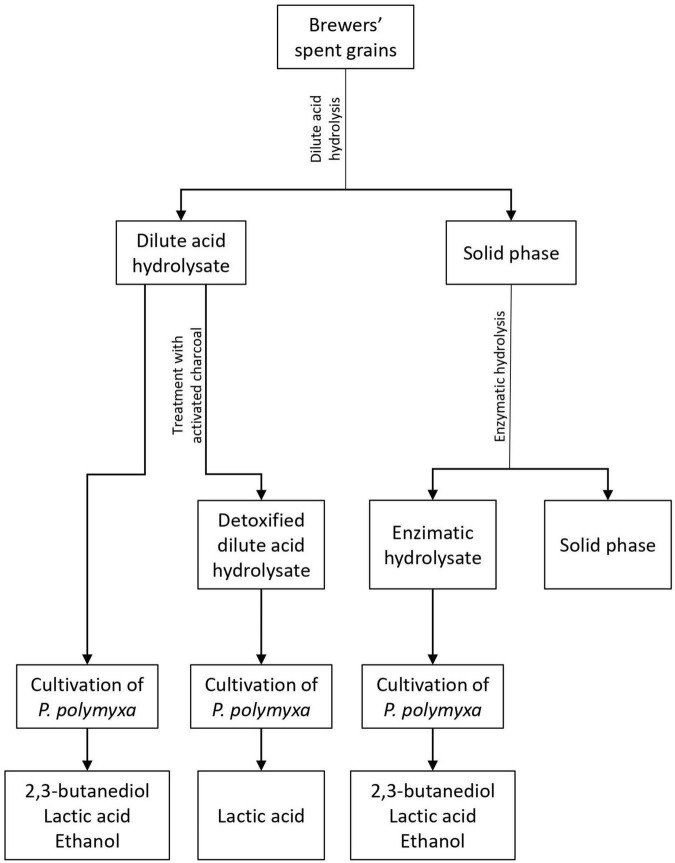
Graphical representation of plan and performance of experiments.

The aim of this work was to define the potential of *P. polymyxa* DSM 742 for sustainable biochemical production. The BSG was selected as a renewable feedstock, and therefore it was hydrolyzed by using dilute sulfuric acid to obtain fermentable sugars. After hydrolysis, the liquid phase of pretreated BSG was detoxified with activated carbon in order to remove bacterial growth inhibitors. The solid phase of pretreated BSG was hydrolyzed with commercial enzymes to maximize the efficiency of fermentable sugar production from this feedstock. In the first stage, the new strain of *P. polymyxa* DSM 742 was cultivated in media with different carbon sources (glucose, xylose, or their mixture) in order to define its potential for 2,3-BD and ethanol or lactate production. In the second stage, *P. polymyxa* was cultivated in the stirred tank bioreactor on the three types of pretreated BSG to define bacterium capacity to use pretreated BSG as a carbon source for 2,3-BD and ethanol or lactate (Figure 1).

## Materials and Methods

### Pretreatment of Brewers’ Spent Grains

The raw material used for pretreatment was BSG obtained after beer production in the microbrewery of the Laboratory for Biochemical Engineering, Industrial Microbiology and Malting and Brewing Technology, Faculty of Food Technology and Biotechnology, University of Zagreb (Zagreb, Croatia). The material after brewing was dried at 105°C to constant weight and stored in a cool and dry place until further use. Dry BSG contained the following (in %): glucan 40.50, xylan 6.14, arabinan 6.32, soluble lignin 5.50, insoluble lignin and ash 26.22, acetate 1.66, formate 1.35, and furans 2.07. The BSG composition was determined by the NREL method (Sluiter et al., 2012).

Dry BSG (1 kg of BSG was suspended in 10 l of 0.5% w/w H_2_SO_4_) was hydrolyzed in the high-pressure reactor (Rosing, Zagreb, Croatia) at 180°C during 10 min, as described in Marđetko et al. (2018). After dilute acid hydrolysis, 6.8 l of the liquid phase and 170 g of the solid phase of pretreated BSG were obtained. The composition of the liquid and solid phases of pretreated BSG was determined as described in Marđetko et al. (2018). The obtained liquid phase of pretreated BSG had the following composition (in g l^–1^): glucose 26.89, xylose 11.57, and acetate 0.77. The liquid phase of pretreated BSG was additionally treated with activated charcoal (Merck KGaA, Darmstadt, Germany) to remove toxic compounds [18 g activated charcoal/1 l of liquid phase at 20°C for 1 h and at a magnetic shaker rotation speed of 250 rpm] (Sampaio et al., 2006; Canilha et al., 2008). Activated charcoal was removed by filtration on a Büchner funnel (filter paper LabExpert, pore size 55 mm, Hilden, Germany). The obtained detoxified liquid phase of pretreated BSG had the same carbohydrate composition as the liquid phase of pretreated BSG prior to detoxification. Both the original liquid phase and detoxified liquid phase of pretreated BSG were used for cultivations of *P. polymyxa* DSM 742.

The composition of the solid phase of pretreated BSG was as follows (in %): glucan 36.69, xylan 2.13, acid insoluble lignin and ash 47.49, and moisture 13.69. The solid phase of pretreated BSG (350 g, obtained from several batches performed in the same pretreatment conditions) was suspended in 5 l of 0.05 mol l^–1^ acetate buffer (pH 5.0), and two commercial enzyme cocktails, Viscozyme L (Novozymes, Bagsvaerd, Denmark) at a concentration of 5% vol vol^–1^ of suspension, and Cellulase SAE0020 (Sigma-Aldrich, St. Louis, MO, United States) at a concentration of 2% vol vol^–1^, were added. Viscozyme L [≥100 FBG/g of carboxymethyl cellulose] is a blend of β-glucanases, pectinases, hemicellulases, and xylanases, while Cellulase SAE0020 (≥1,000 FPU/g of cellulose) is a blend of celluloses, β-glucosidases, and hemicellulases. Hydrolysis was performed in a horizontal rotating tubular bioreactor (HRTB; Rosing, Zagreb, Croatia) during 30 h at 40°C and HRTB rotation of 20 rpm. HRTB is made of stainless steel whose interior is characterized by two paddles to homogenize the content of the bioreactor. It is also equipped with temperature and pressure control units, as well as an electric motor and a base unit. The total volume of HRTB is 30 l, and the maximal rotation speed is 60 rpm. After hydrolysis, suspension was filtrated, and 160 g of non-hydrolyzed material and 5 l of hydrolysate were obtained. The obtained hydrolysate of pretreated BSG was used for cultivation of *P. polymyxa* in the stirred tank bioreactor.

### Microorganism and Culture Media

*Paenibacillus polymyxa* DSM 742 was purchased from the German Collection of Microorganisms and Cell Cultures GmbH (Leipzig, Germany). The culture was maintained and propagated regularly in medium with the following composition (in g l^–1^): peptone 5, meat extract 3, and glucose 1. For the preparation of solid media, agar in a concentration of 15 g l^–1^ was added. Inoculum for cultivations was prepared in the same liquid medium.

Media used for cultivations in Erlenmeyer flasks were reported in Schilling et al. (2020) and were of the following composition (in g l^–1^): yeast extract 5, tryptone 5, MgSO_4_ × 7 H_2_O 0.2, KH_2_PO_4_ 3.5, K_2_PO_4_ 2.5, NH_4_COOH 5, and (NH_4_)_2_SO_4_ 4. The solution of trace elements was prepared separately, filtrated through a disposable sterile syringe filter (pore size 0.20 μm, pa 20/25, nylon; CHROMAFIL Xtra, Macherey-Nagel GmbH & Co. KG, Düren, Germany), and added to the media after sterilization. The solution of trace elements contained the following (in g l^–1^): FeSO_4_ × 7 H_2_O 2.5, C_4_H_4_Na_2_O_6_ × 2 H_2_O 2.1, Cl_2_Mn × 2 H_2_O 1.8, CoCl_2_ × 6 H_2_O 0.075, CuSO_4_ × 5 H_2_O 0.031, boric acid 0.258, NaMoO_4_ × 2 H_2_O 0.023, and ZnCl_2_ 0.021. Solutions of carbon sources, 500 g l^–1^ glucose and 500 g l^–1^ of xylose, were prepared separately, sterilized, and added to the culture media in different quantities, depending on the experimental setup. The list of media used for cultivations of *P. polymyxa* DSM 742 is shown in Table 1. All chemicals used in this work were of p.a. purity and purchased from Sigma-Aldrich (St. Louis, MO, United States) if not stated otherwise.

**TABLE 1:** Initial concentration of main carbon sources in media used for cultivations of *P. polymyxa* DSM 742.

Media*	Carbon source concentration (g l^–1^)	
	Glucose	Xylose
glc_30_	30	–
glc_50_	50	–
glc_70_	70	–
xyl_5_	–	5
xyl_10_	–	10
xyl_20_	–	20
xyl_30_	–	30
xyl_50_	–	50
xyl_70_	–	70
glc_10_xyl_10_	10	10
glc_20_xyl_20_	20	20
glc_30_xyl_30_	30	30
glc_27_xyl_12_	27	12
glc_22_xyl_10_	22	10
glc_33_xyl_4_	33	4

*All media are supplemented with yeast extract, salts, and trace elements solution as described in section “Microorganism and Culture Media”.

### Cultivation of *Paenibacillus polymyxa* DSM 742 in Media With Glucose, Xylose, or a Mixture of Glucose and Xylose in Erlenmeyer Flasks

The bacterium *P. polymyxa* DSM 742 from slant agar was transferred into 20 ml of medium for maintenance. The bacterium was cultivated in a rotary shaker (speed of 130 rpm) overnight at 30°C. 10 ml of culture was transferred into 100 ml of the same media to grow overnight in the same conditions. For the study of biomass growth and kinetics of 2,3-BD production, complex media were prepared with glucose and xylose as carbon source and the mixture of both sugars (Table 1) was used. 7.5 ml of previously prepared culture was inoculated in 150 ml of media for Erlenmeyer flask cultivation. Bacterial cultivations were carried out up to 48 h at 30°C with a shaker rotation speed of 130 rpm. 5 ml of the sample was removed each hour. The optical density of biomass was determined on a spectrophotometer (Carry 100, Agilent Technologies, Santa Clara, CA, United States) at 600 nm, while the chemical content of the broth samples was determined by ultra-performance liquid chromatography (UPLC) analysis (Agilent 1290 Infinity II LC System, Santa Clara, CA, United States) after centrifugation (5 min/8,000 rpm). All experiments in Erlenmeyer flasks were done in triplicate, and standard deviations are presented as error bars in figures. The standard deviation of experimental data was calculated by using the standard procedure in the software Statistica 12.0 (StatSoft, Tulsa, OK, United States).

### Cultivation of *Paenibacillus polymyxa* on Different Hydrolysates of Pretreated Brewers’ Spent Grains in the Stirred Tank Bioreactor

For the cultivation of *P. polymyxa* DSM 742 in the stirred tank bioreactor (Biostat Cplus, Sartorius Stedim Biotech GmbH, Göttingen, Germany), the hydrolysates of pretreated BSG with 5 g l^–1^ of yeast extract were used. The carbohydrate composition of the obtained hydrolysates of pretreated BSG is presented in Table 2. The working volume of the bioreactor was 5.5 l, and it was inoculated with 7% (vol/vol) of previously prepared bacterial culture. Cultivations of *P. polymyxa* DSM 742 in the bioreactor were performed at 30°C, pH = 6.5 with pH control (addition of 3 M NaOH or 2 M H_2_SO_4_), and a stirrer rotation speed of 250 rpm, in microaerobic conditions during 96 h. The culture samples were taken each hour in sterile conditions after which optical density was determined by a spectrophotometer (Carry 100, Agilent Technologies, Santa Clara, CA, United States) at 600 nm. The chemical content of the broth sample was determined by UPLC analysis (Agilent 1290 Infinity II LC System, Santa Clara, CA, United States) after centrifugation (5 min/8,000 rpm). All *P. polymyxa* DSM 742 cultivations in the stirred tank bioreactor were repeated, and the average values of all bioprocess parameters are presented in figures.

**TABLE 2:** Carbon source concentrations in different media with pretreated BSG hydrolysates.

Sugar	LP-BSG medium (g l^–1^)	DLP-BSG medium (g l^–1^)	SP-BSG medium (g l^–1^)
Cellobioses	6.97	/	3.69
Glucose	26.89	22.04	33.27
Xylose	11.57	9.67	3.68
Arabinose	5.62	4.73	0.52
Acetate	0.77	0.77	5.65

*LP-BSG, liquid phase of pretreated BSG supplemented with 4 g l^–1^ yeast extract; DLP-BSG, detoxified liquid phase of pretreated BSG supplemented with 4 g l^–1^ yeast extract; SP-BSG, enzymatic hydrolysate of solid phase of pretreated BSG supplemented with 4 g l^–1^ yeast extract.*

### Analytical Methods

Biomass growth during cultivations was monitored by determination of optical density at 600 nm (Carry 100, Agilent Technologies, Santa Clara, CA, United States; Das et al., 2021). The broth samples were centrifuged (see sections “Cultivation of *Paenibacillus polymyxa* DSM 742 in Media With Glucose, Xylose, or a Mixture of Glucose and Xylose in Erlenmeyer Flasks” and “Cultivation of *Paenibacillus polymyxa* on Different Hydrolysates of Pretreated Brewers’ Spent Grains in the Stirred Tank Bioreactor”), and the obtained supernatant was used for UPLC determination of medium compound concentration.

An aliquot of 750 μl of supernatant was mixed with 750 μl of 10% w/w ZnSO_4_. The obtained mixture was vortexed for 20 s and left standing for 10 min, after which the sample was centrifuged at 13,500 rpm during 5 min (CF-10, witeg Labortechnik GmbH, Offenburg, Germany) to remove proteins. A volume of 1.5 ml of supernatant was filtrated through a 0.20-μm nylon syringe filter (Macherey-Nagel GmbH & Co. KG, Düren, Germany) in a vial.

The concentrations of glucose, xylose, lactate, acetate, ethanol, and 2,3-BD in samples were determined by UPLC using the Agilent Technologies 1290 Infinity II LC system (Santa Clara, CA, United States) with a Carbo-H++ precolumn (4 mm × 3 mm; Phenomenex, Des Plaines, IL, United States), Rezex ROA column (15 cm × 7.8 mm; Phenomenex, Des Plaines, IL, United States), and a refractive index detector. The mobile phase was 0.0025 M H_2_SO_4_, the volume of the analyzed sample was 10 μl, and the flow rate was 0.6 ml min^–1^. The temperature of the column was 60°C. The results were analyzed with OpenLab CDS software.

### Bioprocess Efficiency Parameters

Bioprocess efficiency parameters were calculated according to the following equations (the average values of the monitored bioprocess parameters were used in these calculations):


(1) $$Y_{S}=S_{0}-S$$

(2) $$Y_{P}=P-P{}_{0}$$

(3) YP/S=YPYS=P-P0S-0S

(4) $$r_{S}=\frac{d\gamma {}_{P}}{dt}$$

(5) $$\mu =\frac{dOD{}_{600}}{dt}$$

*S*_0_, *P*_0_ – initial concentration of the substrate or product (g l^–1^)

*S*, *P* – final concentration of the substrate or product (g l^–1^)

*Y*_*S*_ – total consumption of substrates (glucose and/or xylose) (g l^–1^)

*Y*_*P*_ – total product yield of 2,3-BD, ethanol, or lactate (g l^–1^)

*Y*_*P/S*_ – conversion coefficient of substrate in the product (g g^–1^)

*r*_*S*_ – maximal consumption rate of the substrate (h^–1^)

*r*_*P*_ – maximal production rate of 2,3-BD, ethanol, or lactate (h^–1^)

μ – specific growth rate of *P. polymyxa* (h^–1^)

*t* – time (h).

## Results

### Growth Kinetics and Biochemicals Production During Cultivation of *Paenibacillus polymyxa* DSM 742 on Media With Glucose, Xylose, or Their Mixture in Erlenmeyer Flasks

**FIGURE 2:**
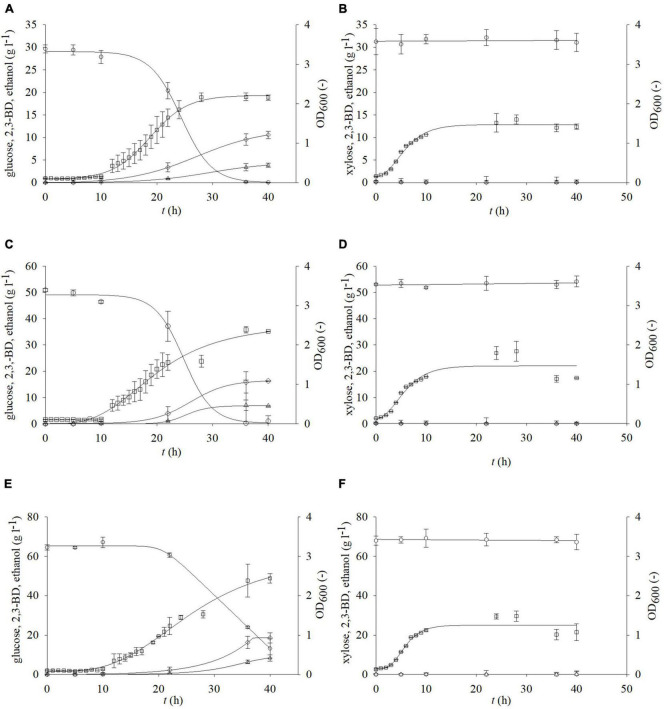
Changes in optical density (OD_600_,□) and concentration of glucose (○) or xylose (⬢), 2,3-BD (⋄), and ethanol (△) during cultivation of bacterium *P. polymyxa* DSM 742 in media with different carbon sources and their concentrations (**A,C,E** – glucose; **B,D,F**—xylose).

**FIGURE 3:**
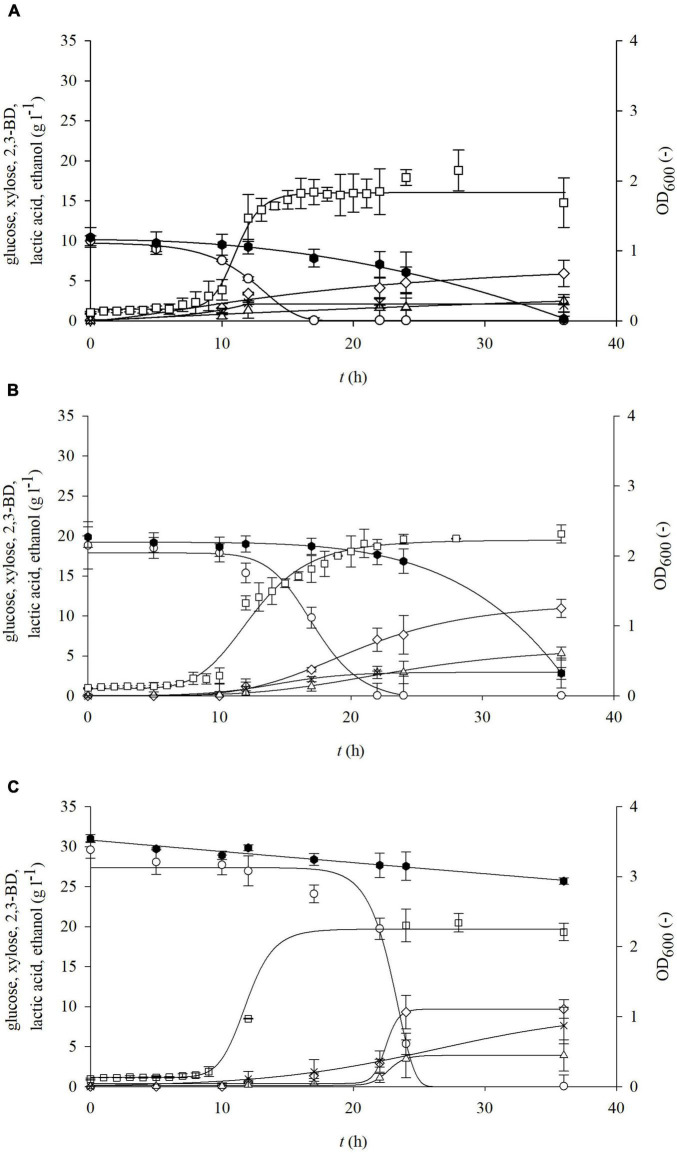
Changes in optical density (OD_600_,□) and concentration of glucose (○), xylose (⬢), 2,3-BD (⋄), lactate (▲), and ethanol (△) during cultivation of bacterium *P. polymyxa* DSM 742 in media with different initial glucose and xylose concentrations [**(A)** 10 g l^–1^ glucose and 10 g l^–1^ xylose; **(B)** 20 g l^–1^ glucose and 20 g l^–1^ xylose; **(C)** 30 g l^–1^ glucose and 30 g l^–1^ xylose].

**FIGURE 4:**
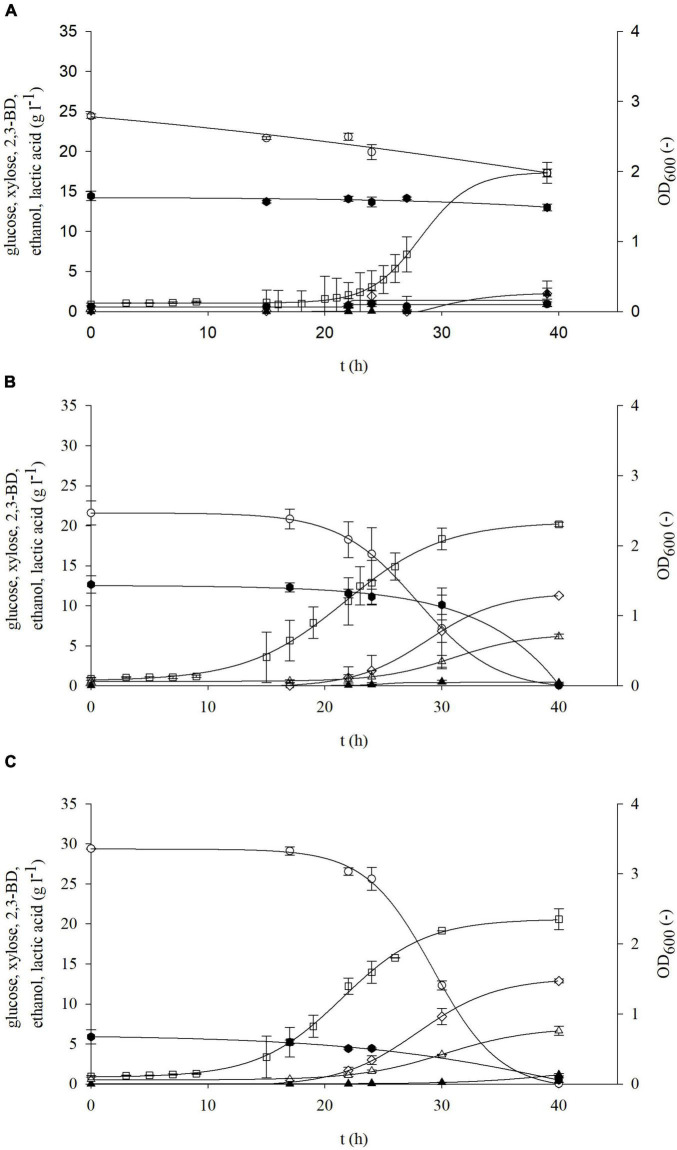
Changes in optical density (OD_600_,□) and concentration of glucose (○), xylose (⬢), 2,3-BD (⋄), lactate (▲), and ethanol (△) during cultivation of bacterium *P. polymyxa* DSM 742 in media with different initial glucose and xylose concentrations [**(A)** 27 g l^–1^ glucose and 12 g l^–1^ xylose; **(B)** 22 g l^–1^ glucose and 10 g l^–1^ xylose; **(C)** 33 g l^–1^ glucose and 4 g l^–1^ xylose].

Glucose and xylose are the main carbohydrates that can be obtained after pretreatment of residual lignocellulosic raw materials. The choice of the pretreatment method greatly influences the concentration of the aforementioned fermentable sugars (Marđetko et al., 2018, 2021). Bacterium *P. polymyxa* can use glucose and xylose for its metabolic needs (Okonkwo et al., 2021). Therefore, it was of great importance to determine the influence of different carbon sources on the growth *P. polymyxa* DSM 742 and production of 2,3-BD and other metabolic products. For this research, different media were prepared, containing glucose and/or xylose as a carbon source. Figure 2 shows the results of cultivation with glucose or xylose as a sole carbon source, while Figures 3, 4 show the results of cultivation with glucose and xylose as carbon sources in nutrient media. As it is shown in Figure 2, the exponential growth phase began after approximately 6 h. The highest rate of glucose consumption was observed between hours 10 and 35 of cultivation with glucose as a sole carbon source (Figure 2 and Table 3). During the cultivation in the media that had an initial glucose concentration of 30 or 50 g l^–1^, all glucose was consumed after 36 h of the bioprocess. The cultivation had an initial glucose concentration of 70 g l^–1^; approximately 18% of the carbon source remained (13.2 g l^–1^) at the end of the cultivation. The biomass concentration was evaluated as optical density determined at 600 nm (OD_600_) according to Das et al. (2021). Furthermore, optical density was also used for bacterial growth rate determination according to Koller et al. (2019). The maximum values of optical density (OD_600_) achieved at the end of *P. polymyxa* DSM 742 cultivation were 1.790 for initial glucose concentration of 30 g l^–1^, 1.589 for 50 g l^–1^, and 1.663 for 70 g of l^–1^, respectively. The determined specific growth rates were 0.318 h^–1^ for 30 g l^–1^, 0.219 h^–1^ for 50 g l^–1^, and 0.216 h^–1^ for 70 g l^–1^ glucose.

**TABLE 3:** Specific growth rate, consumption of substrate and synthesis of product, and yield of cultivation of *P. polymyxa* DSM 742 on media with different carbon sources.

Type of media	μ [h^–1^]	*r*_S glc_ [h^–1^]	*r*_S xyl_ [h^–1^]	*r*_BD_ [h^–1^]	*r*_EtOH_ [h^–1^]	*Y*_P/S BD_ [g g^–1^]	*Y*_P/S EtOH_ [g g^–1^]
Glucose 30 g l^–1^	0.318	0.341	/	0.102	0.109	0.012	0.130
Glucose 50 g l^–1^	0.219	0.384	/	0.120	0.154	0.007	0.135
Glucose 70 g l^–1^	0.216	0.053	/	0.130	0.162	0.005	0.164
Xylose 5 g l^–1^	0.136	/	0.036	0.028	0.022	0.189	0.220
Xylose 10 g l^–1^	0.124	/	0.244	0.088	0.045	0.262	0.166
Xylose 20 g l^–1^	0.074	/	/	/	/	/	/
Xylose 30 g l^–1^	0.204	/	/	/	/	/	/
Xylose 50 g l^–1^	0.213	/	/	/	/	/	/
Xylose 70 g l^–1^	0.184	/	/	/	/	/	/
Glucose (10 g l^–1^) + xylose (10 g l^–1^)	0.297	0.778	0.248	0.840	/	0.291	0.122
Glucose (20 g l^–1^) + xylose (20 g l^–1^)	0.291	1.056	0.137	0.356	/	0.304	0.148
Glucose (30 g l^–1^) + xylose (30 g l^–1^)	0.220	0.410	/	0.256	/	0.277	0.112
Glucose (27 g l^–1^) + xylose (12 g l^–1^)	0.241	0.008	/	0.042	0.022	0.041	0.104
Glucose (22 g l^–1^) + xylose (10 g l^–1^)	0.093	0.083	0.277	0.124	0.107	0.331	0.181
Glucose (33 g l^–1^) + xylose (4 g l^–1^)	0.154	0.393	0.128	0.108	0.097	0.370	0.191

*μ, specific growth rate; r_S glc_, glucose consumption rate; r_S xyl_, xylose consumption rate; r_BD_, production rate for 2,3-BD; r_EtOH_, production rate for ethanol; Y_P/S BD_, product yield for 2,3-BD; Y_P/S EtOH_, product yield for ethanol.*

At the initial glucose concentration of 30 g l^–1^, the maximal 2,3-BD concentration was 10.57 g l^–1^, while the ethanol concentration was 3.85 g l^–1^. A 53.8% (16.26 g l^–1^) of more 2,3-BD was produced in media with an initial glucose concentration of 50 g l^–1^ compared to the media with 30 g l^–1^ and 43% more ethanol than with 30 g l^–1^ (6.75 g l^–1^). The highest concentration of 2,3-BD (18.61 g l^–1^) was obtained during cultivation with 70 g l^–1^ of glucose, which is 12.62% more than 50 g l^–1^ and 42.2% more than 30 g l^–1^. Since xylose is also present in the pretreated BSG, the growth kinetics and synthesis of products on media with xylose were also determined. The highest OD_600_ value (1.781) was observed with an initial xylose concentration of 50 g l^–1^ after 28 h of cultivation (Figure 2). With 30 g l^–1^ of xylose, in the same hour of the bioprocess, 13% lower value (1.536) was observed in comparison with 50 g l^–1^ of xylose, while the lowest concentration of biomass was observed with 70 g l^–1^ of the initial xylose concentration (1.416). During *P. polymyxa* cultivation on media with 5, 10, and 20 g l^–1^ of xylose, slightly lower optical densities were observed than in previous experiments (data not shown). In these cases, the following concentrations of 2,3-BD were obtained: 5 g l^–1^ of xylose (1 g l^–1^ 2,3-BD), 10 g l^–1^ (2.92 g l^–1^ 2,3-BD), and 20 g l^–1^ (0.34 g l^–1^ 2,3-BD). At the same time, the ethanol concentrations were 1.16 g l^–1^ for 5 g l^–1^ of xylose, 1.84 g l^–1^ for 10 g l^–1^, and 0.89 g l^–1^ for 20 g l^–1^, respectively. The increase in initial xylose concentration (5–70 g l^–1^) in medium did not show a clear impact on the bioprocess efficiency parameters (Table 3), although a slight increase in medium optical density was observed. Furthermore, the xylose consumption during *P. polymyxa* DSM 742 cultivation was also at relatively low levels and therefore no significant formation of products (such as 2,3-BD or ethanol) was observed. Application of physiochemical pretreatment methods such as pretreatment of lignocellulosic raw materials in a high-pressure reactor using dilute acid (Marđetko et al., 2018) allows to obtain liquid hydrolysates containing predominantly carbohydrates such as glucose and xylose and some other sugars (e.g., arabinose, galactose or mannose) in significantly smaller (or negligible) concentrations. As hydrolysates of lignocellulosic feedstocks contain multiple carbon sources, it is also necessary to investigate the growth kinetics of *P. polymyxa* in such media. The initial concentrations of both sugars (glucose and xylose) were therefore adjusted so that each medium contains the defined sugar concentration (Table 1 and Figures 3, 4). The selected sugar concentrations are similar to concentrations in the hydrolysate of lignocellulosic raw materials.

During bacterial cultivation in media with 10 g l^–1^ glucose and 10 g l^–1^ xylose and with 20 g l^–1^ glucose and 20 g l^–1^ xylose, the exponential phase started after 7 h of cultivation, while in media with 30 g l^–1^ glucose and 30 g l^–1^ xylose it started after 9 h (Figure 3). After 17 h of cultivation with 10 g l^–1^ glucose and xylose, glucose was completely exhausted, while xylose was depleted after 40 h. In media with 20 g l^–1^ of each sugar, glucose was consumed after 24 h, while 14% (2.82 g l^–1^) of the initial concentration of xylose remained at the end of cultivation. Glucose was also completely exhausted in the media with an initial concentration of 30 g l^–1^ after 24 h, while at the end of cultivation, 85% of the initial xylose concentration (25.68 g l^–1^) remained. The rate of glucose uptake (Table 3) is 2–3 times higher in media with both carbon sources than in substrates containing only glucose. Also, in contrast to the media which contain only xylose, when both carbon sources are present, xylose is partially exhausted especially at lower initial concentrations (10 and 20 g l^–1^). During bacterial cultivation on media with non-equal glucose-to-xylose ratios (similar to the BSG hydrolysates), a similar bioprocess behavior (Figure 4) was observed as in the previous set of experiments. As it is shown in Figure 4A, both sugars were not consumed during bacterial cultivation at 27 g l^–1^ glucose and 12 g l^–1^ of xylose in the period of 40 h probably due to the prolonged bacterial lag phase (cca. 20 h). In other two cases (Figures 4B,C), the bacterial lag phase was shorter (cca. 10 h) and consequently both sugars were consumed in the 40-h period. In both sets of the experiment, it was observed that *P. polymyxa* DSM 742 consumed sugars sequentially. The consumption of xylose started when glucose was almost exhausted.

During the cultivation of *P. polymyxa* DSM 742 on media with combination of the two carbon sources, the following products were obtained: 2,3-BD, ethanol, and lactate. As expected, the highest concentrations of products were determined at the end of the bioprocess. Synthesis of products starts at the beginning of the exponential phase and continues during the stationary bacterial growth phase. The highest concentration of 2,3-BD (10.95 g l^–1^) was achieved in media with an equal glucose-to-xylose ratio at the combination of 20 g l^–1^ of glucose and 20 g l^–1^ xylose (Figure 3B). In media with 10 g l^–1^ of the initial concentration, the highest obtained concentration of 2,3-BD was 5.92 g l^–1^, while 9.70 g l^–1^ of 2,3-BD was produced with 30 g l^–1^, which is 50 and 22% less compared to the media with 40 g l^–1^ of total sugars, respectively. Compared to cultivations performed on glucose as the only carbon source, the specific production rate of 2,3-BD (Table 3) is multiple times higher: 0.840 h^–1^ (10 g l^–1^ glucose and 10 g l^–1^ xylose), 0.356 h^–1^ (20 g l^–1^ glucose and 20 g l^–1^ xylose), and 0.256 h^–1^ (30 g l^–1^ glucose and 30 g l^–1^ xylose). Except 2,3-BD, as a product of cultivation, ethanol was also determined in samples. In cultivation with 10 g l^–1^ glucose and xylose (Figure 3A), the ethanol concentration of 2.48 g l^–1^ was achieved, while twice as much (5.32 g l^–1^) was achieved in media with 20 g l^–1^ sugars. The increased total sugar concentration (30 g l^–1^ glucose + 30 g l^–1^ xylose; Figure 3C) yielded 26.6% less ethanol in comparison with the media with 20 g l^–1^ (Figure 3B), but 57% more than on the media with 10 g l^–1^ (Figure 3A). The highest concentration of lactate (7.63 g l^–1^) was produced in media with 30 g l^–1^ of glucose and xylose (Figure 3C), 62.6% less (2.85 g l^–1^) lactate was produced with 20 g l^–1^ of glucose and xylose (Figure 3B), while 1.94 g l^–1^ (74.6 % less) of lactate was produced in media containing 10 g l^–1^ of glucose and xylose (Figure 3A). During the cultivation of *P. polymyxa* DSM 742 in media with a non-equal glucose-to-xylose ratio, the highest 2,3-BD concentration (12.88 g l^–1^) was observed in medium with 33 g l^–1^ of glucose and 4 g l^–1^ of xylose together with the ethanol concentration of 6.63 g l^–1^ and lactate concentration of 1.16 g l^–1^ (Figure 4C). A slightly lower 2,3-BD concentration (11.26 g l^–1^) was obtained during bacterial cultivation on the medium with 22 g l^–1^ of glucose and 10 g l^–1^ of xylose together with the ethanol concentration of 6.16 g l^–1^ and lactate concentration of 0.36 g l^–1^ (Figure 4B). In this set of experiments, the lowest 2,3-BD concentration (2.40 g l^–1^) was observed during bacterial cultivation in medium with 27 g l^–1^ of glucose and 12 g l^–1^ of xylose together with the ethanol concentration of 0.92 g l^–1^ and lactate concentration of 2.22 g l^–1^ (Figure 4A). It is known in literature that bacteria *P. polymyxa* can also produce lactate during cultivation (Dai et al., 2014; Cao et al., 2017; Okonkwo et al., 2020). In our research, lactate was not determined during cultivation with glucose or xylose as a sole carbon source, but when two carbon sources were used, lactate production was observed. Also, it can be observed that the concentration of produced lactate increases with the increase of the total initial sugar concentration by an equal glucose-to-xylose ratio in cultivation media. However, the non-equal ratio of glucose and xylose (2.25:1, 2.2:1, and 8.25:1) in media is related to the considerably lower lactate production during *P. polymyxa* DSM 742 cultivations. Therefore, further research is required to define the optimal glucose-to-xylose ratio for *P. polymyxa* DSM 742 cultivation for 2,3-BD production.

### Growth Kinetics and Biochemicals Production During Cultivation of *Paenibacillus polymyxa* DSM 742 on Different Brewers’ Spent Grains Hydrolysates in a Stirred Tank Bioreactor

**FIGURE 5:**
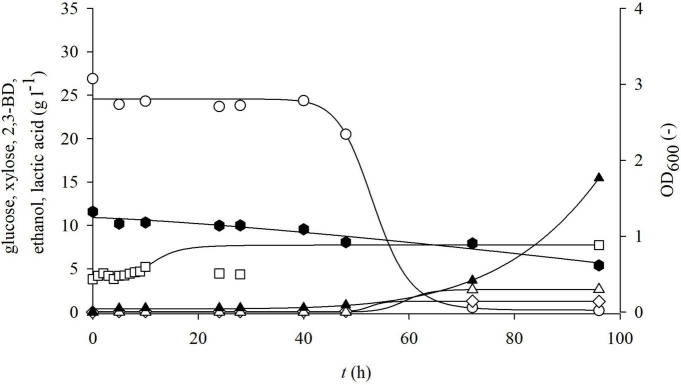
Changes in optical density (OD_600_,□) and concentration of glucose (○), xylose (⬢), 2,3-BD (⋄), ethanol (△), and lactate (▲) during cultivation of bacterium *P. polymyxa* DSM 742 in media with liquid phase of dilute acid BSG hydrolysate in the stirred tank bioreactor.

**FIGURE 6:**
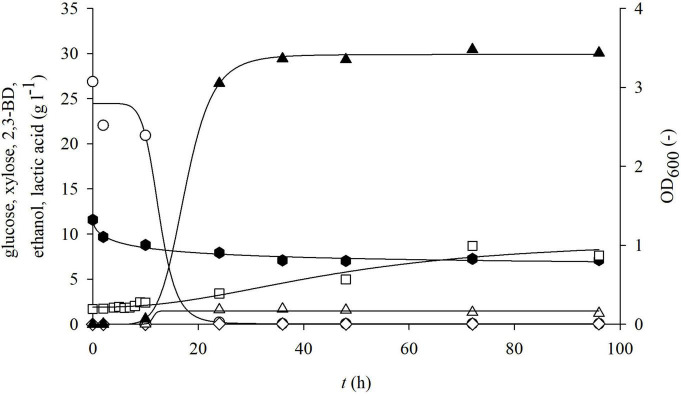
Changes in optical density (OD_600_,□) and concentration of glucose (○), xylose (⬢), 2,3-BD (⋄), ethanol (△), and lactate (▲) during cultivation of bacterium *P. polymyxa* DSM 742 in media with liquid phase of detoxified dilute acid BSG hydrolysate in the stirred tank bioreactor.

**FIGURE 7:**
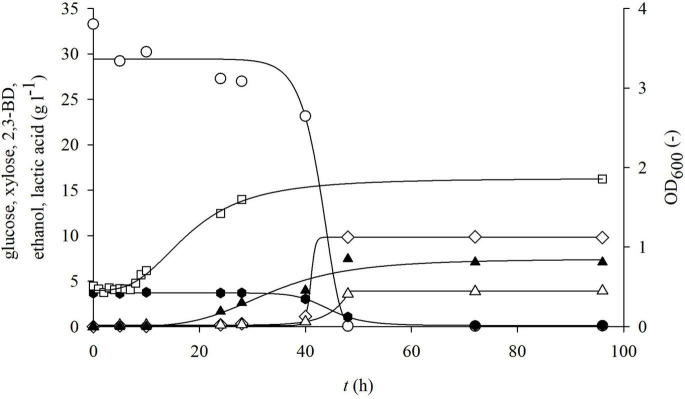
Changes in optical density (OD_600_,□) and concentration of glucose (○), xylose (⬢), 2,3-BD (⋄), ethanol (△), and lactate (▲) during cultivation of bacterium *P. polymyxa* DSM 742 in media with enzymatically hydrolyzed solid phase of dilute acid BSG hydrolysate in the stirred-tank bioreactor.

In the first part of this research, the main focus was to determine growth kinetics and biochemical production during cultivation of *P. polymyxa* DSM 742 in media with glucose and/or xylose, that is, carbohydrates that can be obtained after dilute acid pretreatment of BSG. Physiochemical and enzymatic pretreatment methods, as described in “Materials and Methods,” yielded three different cultivation media. First, BSG were hydrolyzed with dilute acid (0.5% w/w H_2_SO_4_) in a high-pressure reactor, using the method by Marđetko et al. (2018), in order to obtain a liquid hydrolysate rich in fermentable sugars and a solid part in which remain non-hydrolyzed complex sugars. The obtained liquid phase of pretreated BSG was detoxified with active coal in order to remove possible inhibitory compounds, and thus second cultivation media were obtained. The solid part of pretreated BSG was hydrolyzed with commercially available lignocellulolytic enzymes to produce third cultivation media. After obtaining the media, batch cultivation of *P. polymyxa* DSM 742 in a stirred tank bioreactor was conducted on non-detoxified and detoxified liquid phases and an enzymatic hydrolyzed solid phase of pretreated BSG. The results are shown on Figures 5–7.

From the obtained results, it can be observed that the adaptation phase of *P. polymyxa* to non-detoxified, detoxified, and enzymatic hydrolysates of BSG was approximately the same and lasted around 10 h. During the cultivation of *P. polymyxa* DSM 742 on the non-detoxified liquid phase of pretreated BSG (Figure 5), it was observed that glucose was completely consumed after 72 h of cultivation and the maximum consumption rate was 0.127 h^–1^. Xylose was consumed slower than glucose, so a final concentration of xylose was 46.5% of the initial concentration (11.58 to 5.38 g l^–1^) with a specific consumption rate of 0.0089 h^–1^. The highest concentration of 2,3-BD was reached after 72 h as well as maximal ethanol concentration. The majority of ethanol was produced during first 48 h, while its concentration was slightly increased with prolonged cultivation. After 72 h of cultivation, 1.26 g l^–1^ of 2,3-BD, 2.5 g l^–1^ of ethanol, and 3.6 g l^–1^ of lactate were produced. At the end of cultivation (96 h), there was a decrease in the concentration of 2,3-BD (1.22 g l^–1^), while the concentrations of other metabolic products increased (15.43 g l^–1^ of lactate and 2.61 g l^–1^ of ethanol).

The results of cultivation of the bacterium *P. polymyxa* DSM 742 in the liquid detoxified phase of pretreated BSG are shown in Figure 6. The initial total phenol concentration in the liquid phase of pretreated BSG was 1.40 g l^–1^, but after the detoxification process it was only 0.40 g l^–1^. As it is shown in Figure 6, glucose is completely exhausted until the end of the exponential phase, while the concentration of xylose at the end of the bioprocess was 61.5% of the initial concentration (decrease from 11.58 to 7.12 g l^–1^). After 24 h of cultivation, 26.7 g l^–1^ of lactate was produced, which makes 87.7% of the total synthesis. At the end of the bioprocess, at 96 h, 30.07 g l^–1^ lactate and 3.96 g l^–1^ acetate were synthetized on the detoxified pretreated BSG.

After separation at the end of the physiochemical pretreatment, the solid part of lignocellulosic raw materials is usually discarded as a waste (Macheiner et al., 2003). The solid phase of pretreated BSG, after pretreatment in our research, contains 36.69% w/w of glucans and 2.13% w/w of xylans, meaning that it still has complex sugars that can be used for obtaining fermentable sugars. Therefore, the solid part of pretreated BSG was enzymatically hydrolyzed in acetic buffer in the HRTB. After enzymatic treatment, 33.27 g l^–1^ of glucose and 3.68 g l^–1^ of xylose were obtained, thus increasing the overall carbohydrate yield from BSG. During cultivation in such media, it was observed that the lag phase was approximately 7 h (Figure 7). The maximal specific glucose consumption rate was 0.758 h^–1^ (Table 4). After 48 h of cultivation, products that were determined were 9.84 g l^–1^ 2,3-BD, 3.58 g l^–1^ ethanol, and 7.45 g l^–1^ lactate. The specific product concentration did not significantly change with prolongation of cultivation.

**TABLE 4:** Bioprocess efficiency parameters during cultivation of bacterium *P. polymyxa* DSM 742 on media with different BSG hydrolysates.

Type of media	μ [h^–1^]	*r*_S glc_ [h^–1^]	*r*_S xyl_ [h^–1^]	*r*_BD_ [h^–1^]	*r*_EtOH_ [h^–1^]	*r*_LA_ [h^–1^]	*Y*_P/S, BD_ [g g^–1^]	*Y*_P/S, EtOH_ [g g^–1^]
LP-BSG	0.0063	0.127	0.0067	0.097	0.17	/	0.254	0.102
DLP-BSG	0.0228	0.235	0.0089	/	/	0.273	0.001	0.000
SP-BSG	0.0528	0.758	0.0958	0.160	0.13	/	0.033	0.070

*μ, specific growth rate; r_S glc_, glucose consumption rate; r_S xyl_, xylose consumption rate; r_BD_, production rate for 2,3-BD; r_EtOH_, production rate for ethanol; r_LA_, production rate for lactate; Y_P/S BD_, product yield for 2,3-BD; Y_P/S EtOH_, product yield for ethanol; LP-BSG, liquid phase of pretreated BSG supplemented with 4 g l^–1^ yeast extract; DLP-BSG, detoxified liquid phase of pretreated BSG supplemented with 4 g l^–1^ yeast extract; SP-BSG, enzymatic hydrolysate of solid phase of pretreated BSG supplemented with 4 g l^–1^ yeast extract.*

## Discussion

In beer production, BSG is generated in large quantities during the whole year. Due to its high protein and carbohydrate content, it can be used as a raw material in biotechnology for the production of phenolic acids, lactate, biogas, bioethanol, or xylitol or as a medium for cultivation of various microorganisms, for production of enzymes, or as a carrier for immobilizing yeast during a beer fermentation. It is most often used as livestock feed, as an additive to products intended for human consumption, or as a raw material for production of construction materials, coal, paper, and energy, but also it has the potential to be used as an adsorbent (Mussatto, 2014). The economy of the production of chemicals like 2,3-BD mostly depends on the used renewable raw material which is also a carbon source. The use of cheap residual materials such as BSG can greatly reduce production costs and thus make the production process economically and environmentally sustainable (Amraoui et al., 2021). Due to its high content of polysaccharides, BSG have a great potential for application in the biotechnological industry (Ferraz et al., 2013; Mussatto, 2014; Paz et al., 2019; Jackowski et al., 2020). In addition to the biobased materials, a major role in the production of biochemicals plays selection of an appropriate microorganism as a producer of biochemicals, biofuels, or biomaterials.

Unlike other bacteria that produce 2,3-BD, for example *Klebsiella* sp., *Serratia marcescens*, *Escherichia coli*, and *Enterobacter aerogenes* (Lee et al., 2015; Schilling et al., 2020; Chu et al., 2021), *P. polymyxa* DSM 742 used in this research has a GRAS status and can also produce various other products such as ethanol, lactate, and acetate (Lal and Tabacchioni, 2009; Okonkwo, 2017; Jeong et al., 2019), thus making this selected microorganism ideal for cultivation and setup biorefinery concept to produce various biochemicals from food or agriculture residues, using different pretreatment methods for raw materials and different cultivation techniques to maximize bioprocess yield and efficiency. To this date, there is not enough research data for this strain of *P. polymyxa* DSM 742 that can qualitatively describe growth kinetics and biochemical production during cultivation of selected strain on sugars stemming from pretreated lignocellulosic raw materials as BSG.

Firstly, for characterization of growth kinetics and biochemicals production by strain of *P. polymyxa* DSM 742, we tested different concentrations of two types of carbon source, glucose and xylose, as well as their combination. It is known that glucose and xylose are main sugars obtained after pretreatment of BSG. In this study, different concentrations of single sugars or their mixture in media were tested (Table 1). These sugar concentration ranges and ratios are selected due to their frequent appearances in the hydrolysates of lignocellulosic raw materials (Carvalheiro et al., 2004; Marwoto et al., 2004; Chetrariu and Dabija, 2020). Obtained results have shown that there is an increase in 2,3-BD production with increase in initial glucose concentration. However, it appears that a high concentration of substrate slightly inhibits the growth of selected strain *P. polymyxa*. The highest 2,3-BD concentration was achieved during cultivation with 70 g l^–1^ of glucose and was 18.61 g l^–1^, and 13% less 2,3-BD was produced with 50 g l^–1^ of glucose, while 44% less was produced with the initial concentration of 30 g l^–1^. Synthesis of ethanol is increased with the increase in concentration of glucose in media as well. The concentration of ethanol during cultivation with 30 g l^–1^ of glucose was 4 g l^–1^, 45% more was produced during cultivation on 50 g l^–1^ and 53% more with 70 g l^–1^ of glucose compared to the cultivation on 30 g l^–1^ (Figure 2 and Table 3). During cultivation in media containing only xylose, there was no significant synthesis of any metabolic product 2,3-BD, ethanol, or lactate. The reason for this could be the more demanding uptake of xylose in bacterial cells compared to the glucose uptake. This means that xylose as the only carbon source has negligible impact on the kinetics of product formation. These results need further investigation since literature shows production of acetate or 2,3-BD depending on the sugar content in medium (Marwoto et al., 2004; Okonkwo et al., 2021).

After determination of growth kinetics and production of various biochemicals on glucose and xylose as the only medium carbon sources, a research was conducted where *P. polymyxa* DSM 742 was cultivated in media with combined glucose and xylose as carbon sources, in the range of concentrations of 20 to 60 g l^–1^ of total sugars. During 48 h, the glucose consumption rate was 2–3 times higher compared to the glucose consumption rate in media with glucose as a sole carbon source. Xylose was only partially consumed, especially in media with lower sugar concentrations. Comparison between media with only one sugar type (2,3-BD and ethanol were produced) and media with both sugars shows that lactate was additionally produced. The highest concentrations of 2,3-BD and ethanol were obtained with 33 g l^–1^ of glucose and 4 g l^–1^ xylose, respectively (12.88 g l^–1^ 2,3-BD and 6.63 g l^–1^ ethanol). Production of lactate increases with the increase in total sugar content in media with equal glucose to xylose ratio and the highest lactate concentration (7.63 g l^–1^) was observed during cultivation with 60 g l^–1^ of total sugar concentration. This phenomenon can be explained by the carbon catabolite repression (CCR) or glucose effect where sugars were sequentially used. Xylose consumption begins when glucose was depleted, which leads to the low 2,3-BD yield and productivity (Li et al., 2015). However, the non-equal glucose-to-xylose ratios (2.25:1, 2.2:1, and 8.25:1; Figure 4) in cultivation media are related to the considerably lower lactate concentrations during *P. polymyxa* DSM 742 cultivations in combination with relatively high 2,3-BD concentrations. Additionally, cultivation of the *P. polymyxa* ATCC 12321 strain in media containing non-equal glucose-to-xylose ratios (5:1, 2:1, 1:2, 1:5) shows that the increase in xylose content was related to a decrease in lactate concentrations (Ma et al., 2018). The final lactate concentrations were at similar levels (the range of 0.5–2.5 g l^–1^) during cultivations of both *P. polymyxa* strains in media with non-equal glucose-to-xylose ratios. A comparison between our study and that of Ma et al. (2018) shows that the acetate concentration was enlarged with the increase in xylose concentration in media with non-equal glucose-to-xylose ratios, but in both studies a similar range of acetate concentrations was observed (up to 1.5 g l^–1^). It is known that acetate kinase activity in xylose-grown cells on xylose medium was higher than that of glucose-grown cells. Furthermore, the activity of phosphoketolase responsible for ethanol synthesis was relatively low (Marwoto et al., 2004). Glucose is metabolized faster than xylose by *P. polymyxa* due to the longer time required for synthesis of enzymes involved in xylose metabolism, and therefore, the lag phase in xylose consumption was observed (Ma et al., 2018). Based on the above discussion, it is obvious that both strains of *P. polymyxa* (ATCC 12321 and DSM 742) show a similar metabolic behavior on the media containing glucose and xylose as carbon sources.

The diversity of *P. polymyxa* DSM 724 metabolism can be explained by the fact that 2,3-BD and its precursor acetoin are produced from pyruvate, obtained from sugars and other carbohydrates metabolized either by the glycolytic pathway (hexoses) or *via* a combination with the pentose-phosphate metabolic pathway (e.g., xylose). Acetoin and 2,3-BD are obtained in mixed-acid fermentation, which also generates other final metabolites such as lactate, acetate, succinate, and formate, ethanol, and glycerol. This phenomenon can be explained by the carbon catabolite repression which is a consequence of sequentially sugar consumption in media (Petrov and Petrova, 2021). Depending on the type of producer and the cultivation conditions, these metabolites are obtained in different proportions or do not accumulate at all. The same bacterial strain can produce predominantly 2,3-BD or acetoin as alternative final metabolites *via* the manipulation of bioprocess parameters (Ji et al., 2011; Schilling et al., 2020; Petrov and Petrova, 2021) where one mole of glucose is converted into pyruvate by the simultaneous generation of two moles of ATP and NADH *via* glycolysis. Consequently, two moles of pyruvate are converted into α-acetolactate, which is further decarboxylated to acetoin and finally dehydrogenated to 2,3-BD, while simultaneously one redox equivalent of NAD^+^ is produced. In the absence of alternative electron acceptors during anaerobic and microaerobic conditions, the redox balance is maintained by the formation of additional side products (Schilling et al., 2020) such as lactate and ethanol. Depending on oxygen concentration, lactate dehydrogenase, pyruvate formate lyase, and the pyruvate dehydrogenase complex compete for the intermediate pyruvate. This finally results in the production of lactate, formate, ethanol, or acetate (Alexeeva et al., 2003; Schilling et al., 2020). Pyruvate formate lyase represents a redox valve, which can compensate for the redox imbalance of the 2,3-BD pathway (Adlakha et al., 2015). Due to the redox imbalance of 2,3-BD synthesis, other pathways are required to ensure NAD^+^ equilibrium. When the NAD^+^/NADH balance cannot be restored otherwise under microaerobic conditions, glucose is converted to redox-neutral end products such as ethanol and lactate. Pyruvate formate lyase enables the conversion of pyruvate to acetyl-CoA without generating any redox equivalents. Subsequently, two moles of NAD^+^ are regenerated by the combined action of aldehyde dehydrogenase and alcohol dehydrogenase. This route represents the most efficient pathway for redox regeneration in *P. polymyxa* DSM 742 which allows for the compensation of the redox imbalance of the 2,3-BD biosynthesis (Adlakha et al., 2015; Schilling et al., 2020). From the technological point of view, these results show that it is possible to obtain more than one useful product with multiple carbon sources present in cultivation media as it can be important for a biorefinery approach.

After appropriate pretreatment of BSG, the liquid phase of pretreated BSG containing glucose and xylose can serve as a carbon source for submerged microbial cultivation. Furthermore, the solid phase of pretreated BSG was subsequently hydrolyzed with commercial lignocellulolytic enzymes to maximize the utilization of fermentable sugars from the feedstock. The obtained substrates were used as a carbon source during the cultivation of *P. polymyxa* DSM 742 for the production of various biochemicals such as 2,3-BD, ethanol, and lactate. From the results, it can be seen that glucose was first exhausted while xylose reached 46.5% of the initial concentration at the end of the process. The concentrations of products were lower in comparison with the concentrations reached in media with glucose and/or xylose. Dilute acid-pretreated BSG contain sugars for microbial cultivation but also different chemicals that can inhibit growth of microorganisms and synthesis of products (Bensah and Mensah, 2013; Marđetko et al., 2018). Treatment of the liquid phase of pretreated BSG with active carbon is a cheap and fast way to remove inhibitors. Active carbon can effectively adsorb furfural, hydroxymetilfurfural (HMF), various aromatic compounds, and organic acids and to a very low extent adsorb acetate (Sjulander and Kikas, 2020). During detoxification of poplar prehydrolysates with active carbon (5% w/v), 77.9% of initial furan derivatives and 98.6% of initial aromatic monomers were removed (Zhang et al., 2018). Removal of inhibitors can affect bacterial growth and yield as well as product synthesis (Mhlongo et al., 2015). Therefore, cultivation of *P. polymyxa* DSM 742 was conducted on the detoxified liquid phase of pretreated BSG. As it was expected, until the end of the bioprocess, glucose was completely exhausted, while only 38.5% of the initial xylose concentration was consumed. During this cultivation, 30.07 g l^–1^ lactate and 3.96 g l^–1^ acetate were produced, but production of 2,3-BD and ethanol was negligible. During cultivation of *P. polymyxa* DSM 742 on the non-detoxified liquid phase of pretreated BSG, approximately a twice lower lactate concentration was observed. This phenomenon can be explained by the fact that the metabolism of *P. polymyxa* can be affected by different factors like temperature, pH, concentrations of dissolved oxygen and fermentation inhibitors, and exopolysaccharide synthesis (Okonkwo et al., 2020). 2,3-BD is the main metabolic product at pH range 5.0–6.5 and lactate at pH 7.1–8.0, respectively. This fact shows that the metabolism of *P. polymyxa* can be altered due to the pH level, and consequently the efficiency of 2,3-BD production can be improved by the maintenance of an appropriate pH range in the broth (Hakizimana et al., 2020). During cultivation of *P. polymyxa* DSM 742 in the bioreactor, pH was maintained at pH = 6.5, and therefore enzymes involved in organic acid synthesis (lactate dehydrogenase and acetate kinase) have higher activities and consequently higher lactate and acetate concentrations were observed especially during cultivation on detoxified pretreated BSG. It is known that the acetate concentration in media up to 12 g l^–1^ shows a positive effect on 2,3-BD production (Nakashimada et al., 2000), and therefore it can partially compensate for the 4-hydroxybenzoic acid inhibition effect (it is observed at the concentration of 0.169 g l^–1^) on the 2,3-BD synthesis (Jurchescu, 2014). Adsorption of 4-hydroxybenzoic acid on the active carbon is characterized by a weak bounding (Costa et al., 1988), and consequently, it is can be assumed that its concentration in the detoxified liquid phase of pretreated BSG is still in the inhibitory level. Therefore, 2,3-BD synthesis was on a negligible level during *P. polymyxa* DSM 742 cultivation on this BSG medium.

Recently, it was also discovered that *P. polymyxa* DSM 365 possesses the ability to transform HMF to HMF alcohol and eventually to furoic acid in media with HMF as a sole carbon source. In this study, 95% of HMF was transformed into furoic acid which is further used for notable bacterial growth. The growth of *P. polymyxa* in media with furfural as a sole carbon source was severely inhibited although furfural was converted into furfuryl alcohol (16–50% of the initial concentration), but without notable bacterial growth. Cultivation of *P. polymyxa* DSM 365 on the phenolic compounds as a sole carbon source shows that bacterium has the ability to reduce their concentrations as follows: 94% of p-coumaric acid, 59% of vanillic acid, and 68% vanillin. However, in these experiments bacterial growth was not observed (Okonkwo et al., 2021). It is also known that higher oxygen concentrations have a negative impact on the 2,3-BD production, but positive on the bacterial biomass growth. Our cultivations of *P. polymyxa* DSM 742 were performed in microaerobic conditions due to the fact that relatively low final bacterial biomass concentrations were obtained. During 2,3-BD production, *P. polymyxa* synthesizes the exopolysaccharides (cca 50 g l^–1^) and therefore 2,3-BD production efficiency is reduced. Furthermore, exopolysaccharides can clog bioreactor pipes, increase energy consumption for broth mixing, and considerably complicate the 2,3-BD downstream process (Okonkwo et al., 2020). Based on the previous consideration, it is obvious that different cultivation factors of *P. polymyxa* DSM 742 have to be carefully considered in order to obtain desirable bacterial metabolic products like 2,3-BD, ethanol, or lactate. On the basis of the obtained results, it is clear that different methods of processing the same raw material, BSG, can be used to obtain several different products. This phenomenon is still not well described in literature; therefore, it needs further investigation on metabolic pathways of the *P. polymyxa* DSM 742 strain.

The solid part that remains after dilute acid pretreatment of BSG contains carbohydrates which are not used (for example, glucans are present at 36.69% w/w). Rather than to dispose this as a waste, it can be processed by enzymatic hydrolysis and consequently increase the overall sugar yield of the raw material for different biotechnological processes. This enzymatically obtained nutrient medium contained 23.73% more glucose and 68.2% less xylose than in the liquid phase of pretreated BSG after dilute acid hydrolysis. The glucose consumption rate was the highest from all studied cultivations on obtained pretreated BSG (0.758 h^–1^), which is 69% higher in comparison with the detoxified liquid phase of pretreated BSG (0.235 h^–1^) and 83% higher in comparison with the non-detoxified liquid phase of pretreated BSG (0.127 h^–1^). The maximum concentrations for all products were reached after 48 h of cultivation (2,3-BD 9.84 g l^–1^, ethanol 3.58 g l^–1^, and lactate 7.45 g l^–1^) and did not change significantly until the end of cultivation. The final concentration of lactate (7.08 g l^–1^) in enzymatic hydrolysate was 50% lower (15.43 g l^–1^) in comparison with the lactate concentration obtained on the non-detoxified liquid phase of pretreated BSG and 76% lower (30.07 g l^–1^) than the lactate concentration obtained on the detoxified liquid phase of pretreated BSG. The production rate of 2,3-BD on the enzymatic hydrolysate of the solid phase of pretreated BSG was 1.65 times higher than on the non-detoxified liquid phase of pretreated BSG, but despite that fact, a higher rate of synthesis was achieved on non-detoxified pretreated BSG than on the enzymatic hydrolysate of the solid phase of pretreated BSG for both 2,3-BD (0.254 vs. 0.033 g g^–1^) and ethanol (0.102 vs. 0.070 g g^–1^).

Using different pretreatment methods of residual lignocellulosic materials as BSG, it is possible to obtain nutrient media for microbiological cultivations to produce a range of useful biochemicals. In this study, it was shown that *P. polymyxa* DSM 742 can produce 2,3-BD, ethanol, and lactate with different production rates and product yields. On the basis of the obtained results, it is obvious that the highest impact on the production of different types of biochemicals have working microorganisms, as well as pretreatment (dilute acid hydrolysis or enzymatic hydrolysis) and detoxification methods. The presented concept of biochemical production, using *P. polymyxa* DSM 742, is of great importance for sustainable development of a biochemicals production which could be included in the biorefinery concept. Furthermore, emphasis should be directed toward usage of new and interesting microorganisms that produce various useful biochemicals from different residual food or agricultural residues.

## Data Availability Statement

The raw data supporting the conclusions of this article will be made available by the authors, without undue reservation.

## Author Contributions

All authors listed contributed to the test performance, data analysis, and writing of the manuscript, and they all approved it for the publication.

## Conflict of Interest

The authors declare that the research was conducted in the absence of any commercial or financial relationships that could be construed as a potential conflict of interest.

## Publisher’s Note

All claims expressed in this article are solely those of the authors and do not necessarily represent those of their affiliated organizations, or those of the publisher, the editors and the reviewers. Any product that may be evaluated in this article, or claim that may be made by its manufacturer, is not guaranteed or endorsed by the publisher.

## References

[B1] Adlakha N. Pfau T. Ebenhöh O. Yazdani S. S. (2015). Insight into metabolic pathways of the potential biofuel producer, *Paenibacillus polymyxa* ICGEB2008. *Biotechnol. Biofuels* 8:159. 10.1186/s13068-015-0338-4 26413158 PMC4583153

[B2] Alexeeva S. Hellingwerf K. J. Teixeira de Mattos M. J. (2003). Requirement of ArcA for redox regulation in *Escherichia coli* under microaerobic but not anaerobic or aerobic conditions. *J. Bacteriol.* 185 204–209. 10.1128/JB.185.1.204-209.2003 12486057 PMC141817

[B3] Amraoui Y. Prabhu A. Vivek N. Coulon F. Chandel A. K. Willoughby N. (2021). Enhanced 2,3-butanediol production by mutant *Enterobacter ludwigii* using brewers’ spent grain hydrolysate: process optimization for a pragmatic biorefinery loom. *Chem. Eng. J.* 427:130851. 10.1021/acssuschemeng.1c03951

[B4] Bensah E. Mensah M. (2013). Chemical pretreatment methods for the production of cellulosic ethanol: technologies and innovations. *Int. J. Chem. Eng.* 2013:719607. 10.1155/2013/719607

[B5] Canilha L. Carvalho W. Giulietti M. Felipe M. D. G. A. Almeida E. Silva J. B. (2008). Clarification of wheat straw-derivies medium with ion-exchange resins for xylitol crystallization. *J. Chem. Technol. Biotechnol.* 83 715–721. 10.1002/jctb.1861

[B6] Cao C. Zhang L. Gao J. Xu H. Xue F. Huang W. (2017). Research on the solid state fermentation of Jerusalem artichoke pomace for producing R,R-2,3-butanediol by *Paenibacillus polymyxa* ZJ-9. *Appl. Biochem. Biotechnol.* 182 687–696. 10.1007/s12010-016-2354-7 27943035

[B7] Carvalheiro F. Duarte L. C. Medeiros R. Girio F. G. (2004). Optimization of brewery’s spent grain dilute-acid hydrolysis for the production of pentose-rich culture media. *Appl. Biochem. Biotechnol.* 115:1059. 10.1385/abab:115:1-3:1059 15054253

[B8] Chetrariu A. Dabija A. (2020). Brewer’s spent grains: possibilities of valorization. *Appl. Sci.* 10:5619. 10.3390/app10165619

[B9] Chu W. Jiang K. Lu P. Xu Y. Yang J. Wei X. (2021). Metabolic regulation and optimization of oxygen supply enhance the 2,3-butanediol yield of the novel *Klebsiella* sp. isolate FSoil 024. *Biotechnol. J.* 14:e2100279. 10.1002/biot.202100279 34390606

[B10] Costa E. Calleja G. Marijuán L. (1988). Comparative adsorption of phenol, p-nitrophenol and p-hydroxybenzoic acid on activated carbon. *Ads. Sci. Technol.* 5 213–228. 10.1177/026361748800500304

[B11] Dai J.-J. Cheng J.-S. Liang Y.-Q. Jiang T. Yuan Y.-J. (2014). Regulation of extracellular oxidoreduction potential enhanced (R,R)-2,3-butanediol production by *Paenibacillus polymyxa* CJX518. *Bioresour. Technol.* 167 433–440. 10.1016/j.biortech.2014.06.044 25006018

[B12] Das A. Prakash G. Lali A. M. (2021). 2,3-butanediol production using soy-based nitrogen source and fermentation process evaluation by a novel isolate of *Bacillus licheniformis* BL1. *Prep. Biochem. Biotechnol.* 51 1046–1055. 10.1080/10826068.2021.1894443 33719922

[B13] Daud N. S. Din A. R. J. M. Rosli M. A. Azam Z. M. Othman N. Z. Sarmidi M. R. (2019). *Paenibacillus polymyxa* bioactive compounds for agricultural and biotechnological applications. *Biocatal. Agric. Biotechnol.* 18 92–101. 10.1016/j.bcab.2019.101092

[B14] Ferraz E. Coroado J. Gamelas J. Silva J. Rocha F. Velosa A. (2013). Spent brewery grains for improvement of thermal insulation of ceramic bricks. *J. Mater. Civil Eng.* 25 1638–1646. 10.1061/(ASCE)MT.1943-5533.0000729 29515898

[B15] Grady E. N. MacDonald J. Liu L. Richman A. Yuan Z. C. (2016). Current knowledge and perspectives of *Paenibacillus*: a review. *Microb. Cell Fact.* 1 203–215. 10.1186/s12934-016-0603-7 27905924 PMC5134293

[B16] Hakizimana O. Matabaro E. Lee B. (2020). The current strategies and parameters for the enhanced microbial production of 2,3-butanediol. *Biotechnol. Rep.* 25:e00397. 10.1016/j.btre.2019.e00397 31853445 PMC6911977

[B17] He Z. Kisla D. Zhang L. Yuan C. Green-Church K. B. Yousef A. E. (2007). Isolation and identification of *Paenibacillus polymyxa* strain that coproduces a novel lantibiotic and polymyxin. *Appl. Environ. Microb.* 73 168–178. 10.1128/AEM.02023-06 17071789 PMC1797129

[B18] Ikram S. Huang L. Zhang H. Wang J. Yin M. (2017). Composition and nutrient value proposition of brewers spent grain. *J. Food Sci.* 82 2232–2242. 10.1111/1750-3841.13794 28833108

[B19] Jackowski M. Niedźwiecki Ł Jagiełło K. Uchańska O. Trusek A. (2020). Brewer’s spent grains—valuable beer industry by-product. *Biomolecules* 10:1669. 10.3390/biom10121669 33322175 PMC7764043

[B20] Jeong H. Choi S. Ryu C. Park S. (2019). Chronicle of a soil bacterium: *Paenibacillus polymyxa* E681 as a tiny guardian of plant and human health. *Front. Microbiol.* 10:467. 10.3389/fmicb.2019.00467 30930873 PMC6429003

[B21] Ji X. J. Huang H. Ouyang P. K. (2011). Microbial 2,3-butanediol production: a state-of-the-art review. *Biotechnol. Adv.* 29 3. 10.1016/j.biotechadv.2011.01.007 21272631

[B22] Jurchescu I.-M. (2014). *2,3–Butanediol Production with GRAS Microorganisms – Screening, Cultivation, Optimization and Scale-up.* dissertation. Braunschweig: Fakultät für Lebenswissenschaften der Technischen Universität Carolo-Wilhelmina.

[B23] Kajimura Y. Kaneda M. (1997). Fusaricidins B, C and D, new depsipeptide antibiotics produced by *Bacillus polymyxa* KT-8: isolation, structure elucidation and biological activity. *J. Antibiot.* 50 220–228. 9439693

[B24] Karlović A. Jurić A. Ćorić N. Habschied K. Krstanović V. Mastanjević K. (2020). By-products in the malting and brewing industries - re - usage possibilities. *Fermentation* 6:82. 10.3390/fermentation6030082

[B25] Koller M. Hesse P. Braunegg G. (2019). Application of whey retentate as complex nitrogen source for growth of the polyhydroxyalkanoate producer strain DSM1023. *EuroBiotech J.* 3 78–89. 10.2478/ebtj-2019-0009

[B26] Lal S. Tabacchioni S. (2009). Ecology and biotechnological potential of *Paenibacillus polymyxa*: a minireview. *Indian J. Microbiol.* 49 2–10. 10.1007/s12088-009-0008-y 23100748 PMC3450047

[B27] Lee S. J. Lee J. H. Yang X. Kim S. B. Lee J. H. Yoo H. Y. (2015). Phenolic compounds: strong inhibitors derived from lignocellulosic hydrolysate for 2,3-butanediol production by *Enterobacter aerogenes*. *Biotechnol. J.* 10 1920–1928. 10.1002/biot.201500090 26479290

[B28] Li L. Li K. Wang Y. Chen C. Xu Y. Zhang L. (2015). Metabolic engineering of *Enterobacter cloacae* for high-yield production of enantiopure (2R,3R)-2,3-butanediol from lignocellulose-derived sugars. *Metab. Eng.* 28 19–27. 10.1016/j.ymben.2014.11.010 25499652

[B29] Li Y. Chen S. (2019). Fusaricidin produced by *Paenibacillus polymyxa* WLY78 induces systemic resistance against *Fusarium* wilt of cucumber. *Int. J. Mol. Sci.* 20:5240. 10.3390/ijms20205240 31652608 PMC6829208

[B30] Ma K. He M. You H. Pan L. Wang Z. Wang Y. (2018). Improvement of (R,R)-2,3-butanediol production from corn stover hydrolysate by cell recycling continuous fermentation. *Chem. Eng. J.* 332 361–369. 10.1016/j.cej.2017.09.097

[B31] Macheiner D. Adamitsch B. F. Karner F. Hampel W. A. (2003). Pretreatment and hydrolysis of brewer’s spent grains. *Eng. Life Sci.* 3 401–405. 10.1002/elsc.200301831

[B32] Marđetko N. Novak M. Trontel A. Grubišić M. Galić M. Šantek B. (2018). Bioethanol production from dilute-acid pre-treated wheat straw liquor hydrolysate by genetically engineered *Saccharomyces cerevisiae*. *Chem. Biochem. Eng. Q.* 32 483–499. 10.15255/CABEQ.2018.1409

[B33] Marđetko N. Trontel A. Novak M. Pavlečić M. Didak Ljubas B. Grubišić M. (2021). Screening of lignocellulolytic enzyme activities in fungal species and sequential solid-state and submerged cultivation for the production of enzyme cocktails. *Polymers* 13:3736. 10.3390/polym13213736 34771293 PMC8588072

[B34] Marwoto B. Nakashimada Y. Kakizono T. Nishio N. (2004). Metabolic analysis of acetate accumulation during xylose consumption by *Paenibacillus polymyxa*. *Appl. Microbiol. Biotechnol.* 64 112–119. 10.1007/s00253-003-1435-z 14556038

[B35] McCarthy A. L. O’Callaghan Y. C. Connolly A. Piggott C. O. FitzGerald R. J. O’Brien N. M. (2013). *In vitro* antioxidant and anti-inflammatory effects of brewers’ spent grain protein rich isolate and its associated hydrolysates. *Food Res. Int.* 50 205–212. 10.1039/C3FO60191A 24113874

[B36] Meena V. Mishra P. Bisht J. Pattanayak A. (2017). *Agriculturally Important Microbes for Sustainable Agriculture. Volume I: Plant-Soil-Microbe Nexus.* Berlin: Springer. 10.1007/978-981-10-5343-6

[B37] Mhlongo S. den Haan R. Viljoen-Bloom M. van Zyl W. (2015). Lignocellulosic hydrolysate inhibitors selectively inhibit/deactivate cellulase performance. *Enzyme Microb. Technol.* 81 16–22. 10.1016/j.enzmictec.2015.07.005 26453468

[B38] Moo-Young M. (2011). *Comprehensive Biotechnology.* Amsterdam: Elsevier.

[B39] Mussatto S. I. (2014). Brewer’s spent grain: a valuable feedstock for industrial applications. *J. Sci. Food Agric.* 94 1264–1275. 10.1002/jsfa.6486 24254316

[B40] Nakashimada Y. Marwoto B. Kashiwamura T. Kakizono T. (2000). Enhanced 2,3-butanediol production by addition of acetic acid in *Paenibacillus polymyxa*. *J. Biosci. Bioeng.* 90 661–664. 10.1016/S1389-1723(00)90013-6 16232928

[B41] Okonkwo C. (2017). *Process Development and Metabolic Engineering to Enhance 2,3-Butanediol Production by Paenibacillus polymyxa DSM 365*. dissertation. Columbus, OH: The Ohio State University.

[B42] Okonkwo C. C. Ujor V. Cornish K. Ezeji T. C. (2020). Inactivation of the levansucrase gene in *Paenibacillus polymyxa* DSM 365 diminishes exopolysaccharide biosynthesis during 2,3- butanediol fermentation. *Appl. Environ. Microbiol.* 86:e00196-20. 10.1128/AEM.00196-20 32144108 PMC7170477

[B43] Okonkwo C. C. Ujor V. Ezeji T. C. (2021). Production of 2,3-butanediol from non-detoxified wheat straw hydrolysate: impact of microbial inhibitors on *Paenibacillus polymyxa* DSM 365. *Ind. Crops Prod.* 159:113047. 10.1016/j.indcrop.2020.113047

[B44] Paz A. Outeiriño D. Pérez Guerra N. Domínguez J. M. (2019). Enzymatic hydrolysis of brewer’s spent grain to obtain fermentable sugars. *Bioresour. Technol.* 275 402–409. 10.1016/j.biortech.2018.12.082 30605827

[B45] Petrov K. Petrova P. (2021). Current advances in microbial production of acetoin and 2,3-butanediol by *Bacillus* spp. *Fermentation* 7:307. 10.3390/fermentation7040307

[B46] Sampaio F. C. Lopes Passos F. M. Vieira Passos F. J. De Faveri D. Perego P. Converti A. (2006). Xylitol crystallization from culture media fermented by yeasts. *Chem. Eng. Proc.* 45 1041–1046. 10.1016/j.cep.2006.03.012

[B47] Schilling C. Ciccone R. Sieber V. Schmid J. (2020). Engineering of the 2,3-butanediol pathway of *Paenibacillus polymyxa* DSM 365. *Metab. Eng.* 61 381–388. 10.1016/j.ymben.2020.07.009 32771627

[B48] Sjulander N. Kikas T. (2020). Origin, impact and control of lignocellulosic inhibitors in bioethanol production-a review. *Energies* 13:4751. 10.3390/en13184751

[B49] Sluiter A. Hames B. Ruiz R. Scarlata C. Sluiter J. Templeton D. (2012). *Determination of Structural Carbohydrates and Lignin in Biomass, Technical Report NREL/TP-510-42618.* Golden, CO: National Renewable Energy Laboratory.

[B50] Tang D. Yin G. He Y. Hu S. Li B. Li L. (2009). Recovery of protein from brewer’s spent grain by ultrafiltration. *Biochem. Eng. J.* 48 1–5. 10.1016/j.bej.2009.05.019

[B51] Zhang Y. Xia C. Lu M. Tu M. (2018). Effect of overliming and activated carbon detoxification on inhibitors removal and butanol fermentation of poplar prehydrolysates. *Biotechnol. Biofuels* 11:178. 10.1186/s13068-018-1182-0 29983741 PMC6020205

